# Acute and Postacute Health Care Utilization and Costs After Dengue Infection: A Population-Based Cohort Study

**DOI:** 10.1093/ofid/ofaf401

**Published:** 2025-07-03

**Authors:** Jue Tao Lim, Liang En Wee, Wei Zhi Tan, Calvin Chiew, Lalitha Kurupatham, Cuiqin Poh, Nur-Afidah Md Suhaimi, Hui Zi Chua, Lee Ching Ng, Po Ying Chia, David Chien Boon Lye, Kelvin Bryan Tan

**Affiliations:** National Centre for Infectious Diseases, Singapore, Singapore; Lee Kong Chian School of Medicine, Nanyang Technological University, Singapore, Singapore; National Centre for Infectious Diseases, Singapore, Singapore; Duke-NUS Graduate Medical School, National University of Singapore, Singapore, Singapore; Department of Infectious Diseases, Singapore General Hospital, Singapore, Singapore; National Centre for Infectious Diseases, Singapore, Singapore; National Centre for Infectious Diseases, Singapore, Singapore; Ministry of Health, Singapore, Singapore; Ministry of Health, Singapore, Singapore; Ministry of Health, Singapore, Singapore; Ministry of Health, Singapore, Singapore; Ministry of Health, Singapore, Singapore; National Environment Agency, Singapore, Singapore; National Centre for Infectious Diseases, Singapore, Singapore; Department of Infectious Diseases, Tan Tock Seng Hospital, Singapore, Singapore; National Centre for Infectious Diseases, Singapore, Singapore; Lee Kong Chian School of Medicine, Nanyang Technological University, Singapore, Singapore; Department of Infectious Diseases, Tan Tock Seng Hospital, Singapore, Singapore; Yong Loo Lin School of Medicine, National University of Singapore, Singapore, Singapore; National Centre for Infectious Diseases, Singapore, Singapore; Lee Kong Chian School of Medicine, Nanyang Technological University, Singapore, Singapore; Duke-NUS Graduate Medical School, National University of Singapore, Singapore, Singapore; Ministry of Health, Singapore, Singapore; Saw Swee Hock School of Public Health, National University of Singapore, Singapore, Singapore

**Keywords:** health care utilization, long dengue, postacute dengue

## Abstract

**Background:**

Emerging evidence suggests that postacute sequelae may arise following dengue infection. There has been no quantification of the risk and burden of acute and postacute health care utilization and cost due to dengue.

**Methods:**

We utilized national notification databases from Singapore to construct cohorts of adults first infected with dengue. We compared 55 870 dengue cases with 3 072 309 population-based controls. We estimate excess risks, rates, and burdens of any all-cause inpatient hospital utilization, length of stay in inpatient settings or number of unique hospital inpatient admissions, any all-cause intensive care unit utilization, length of stay in the intensive care unit (ICU) or number of unique ICU admissions, any all-cause emergency department utilization and total number of unique emergency department visits, and any hospitalization costs incurred and excess total hospitalization costs incurred by contrasting dengue patients with population-based controls 0–30 days and 31–300 days following infection.

**Results:**

Dengue patients had elevated risk of emergency department visits, inpatient admissions, and incurring any inpatient costs across the acute and postacute periods. Among patients who had any inpatient admissions, dengue patients were associated with 11.938-fold (95% CI, 10.308–14.179) higher rates of unique inpatient visits, 15.852-fold (95% CI, 11.868–23.861) longer lengths of stay, 1.157-fold (95% CI, 1.123–1.192) higher rates of unique inpatient visits, and 1.339-fold (95% CI, 1.291–1.39) longer lengths of stay in the postacute period. Over the study period, the majority of excess health care costs were estimated to occur in the acute phase (US$21 363 084) compared with the postacute period (US$687 032).

**Conclusions:**

There is increased excess risk and rates of health care utilization in the 300 days post-dengue infection when compared with contemporary population-based controls.

## Graphical Abstract

**Graphical Abstract:**
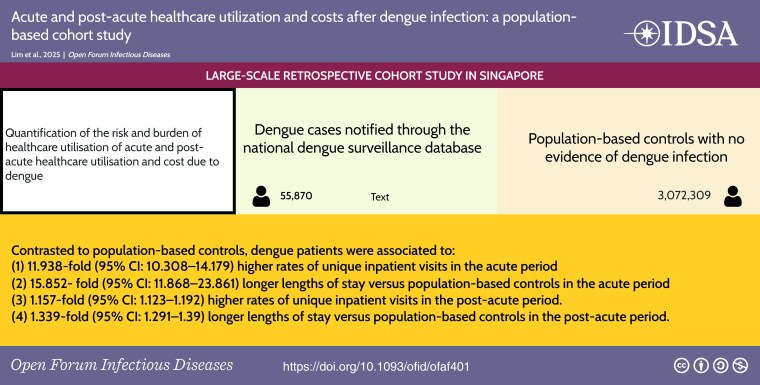
This graphical abstract is also available at Tidbit: https://tidbitapp.io/tidbits/acute-and-post-acute-healthcare-utilization-and-costs-after-dengue-infection-a-population-based-cohort-study-4e39ca6c-de66-4b55-aa31-4188b0165a2c?utm_campaign=tidbitlinkshare&utm_source=ITP

## Teaser

In this population-based cohort study, 30-days post recovery, among dengue patients and population-based controls who had any inpatient admissions, dengue patients were associated to an almost 12-fold higher rates of inpatient visits and 16-fold longer lengths of stay. In the 30-300 days following recovery, dengue patients had 1.157-fold higher rates of unique inpatient visits and 1.339-fold longer lengths of stay in the post-acute period.

Dengue has one of the highest burdens among all vector-borne diseases globally; however, assessment of the economic burden attributable to dengue typically accounts only for morbidity and mortality during the acute phase of disease [1]. Emerging evidence, however, supports chronic symptom persistence and new-onset sequelae following dengue infection in a subset of survivors [2–7]; similar to other viral infections such as COVID-19, in which multisystemic sequelae have been documented to persist years after resolution of acute infection and to translate into excess health care costs [8, 9]. Chronic symptom persistence has been reported in small cohorts of dengue survivors [2, 3]; retrospective cohort studies found increased risk of autoimmune and psychiatric sequelae in dengue survivors vs healthy controls [4–6] and substantially higher risk of postacute sequelae across multiple organ systems following dengue infection, when compared with COVID-19 survivors [7]. However, less is known about the extent to which long-term sequelae following dengue infection translate into increased health care utilization; this is important, given dengue's disproportionate burden in tropical low- and middle-income countries (LMICs), where health care systems are chronically under-resourced.

We therefore sought to estimate the risk and burden of health care utilization up to 300 days following recovery from acute dengue infection, compared with uninfected population-based controls. Outcomes comprised emergency department (ED) visits, all-cause hospital admissions, all-cause intensive care unit admissions, and excess length of stay for admitted individuals and were compared based on previous hospitalization usage, pre- and post-COVID-19 eras of enrollment, comorbidity status, and age.

## METHODS

### Study Setting, Databases and Study Period

**Figure 1.**
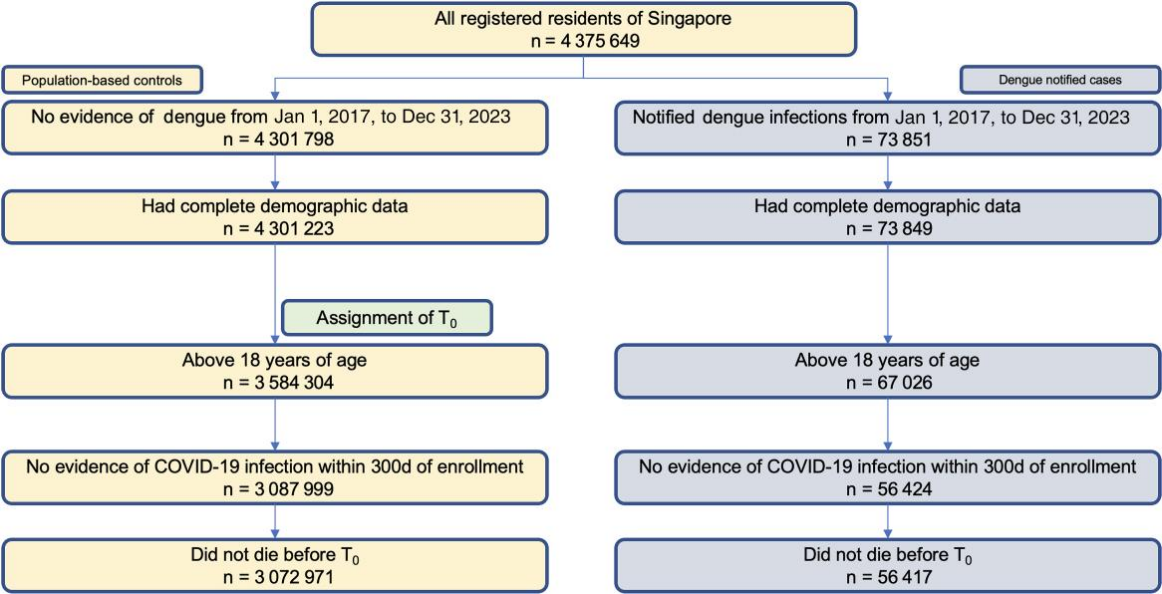
Cohort construction flowchart for comparisons of excess health care utilization in T_0_ to T_0_ + 30 between dengue patients and population-based controls. Abbreviation: COVID-19, coronavirus disease 2019.

Singapore is a multi-ethnic Asian city state (population: 6.03 million). Dengue is endemic in the nation, with nonzero cases reported weekly and at least 3 serotypes documented to be in constant circulation for at least the past 20 years [10]. As for 2024, public health care providers account for almost 70% of the primary care sector, whereas public hospitals handle 90% of the acute hospital workload in Singapore. In Singapore, confirmatory diagnostic testing (eg, NS1 antigen assays, immunoglobulin M enzyme-linked immunoassay) for dengue is widely available across health care settings. Dengue is legally notifiable to the Ministry of Health (MOH) not later than 24 hours from clinical diagnosis or laboratory confirmation [11]. National registries for dengue infection were utilized to construct cohorts first infected with disease from January 1, 2017, to August 31 2023 **(**Figure 1**)**.

### Exposures and Comparators

The primary exposure considered was being test-positive for dengue based on reporting to the national dengue surveillance system. Notified dengue cases were compared against a population-based control group, which included individuals with no evidence of dengue or COVID-19 infection who were Singaporean citizens/permanent residents residing in Singapore for the past 1 year. For population-based controls, we generated a comparator group that had a similar distribution of length of follow-up [12] using exact matching without replacement to match each dengue case with ∼60 population-based Singapore citizens/permanent residents who had no evidence of recorded dengue infection. In matching, the dates of cohort enrollment for the corresponding controls were matched with T_0_ of the dengue-exposed individual—that is, the date of being notified of a dengue exposure (acute dengue group n = 56 417, postacute dengue group n = 55 870). In the control group, we similarly selected individuals who were alive during the first 30 days after the date of enrollment (acute control group n = 3 072 971, postacute control group n = 3 072 309). Participants were followed until August 31, 2023. As the later part of our study period overlapped with the emergence of COVID-19 [13] and long-term sequelae following severe acute respiratory syndrome coronavirus 2 (SARS-CoV-2) infection have been extensively described in our population and elsewhere [8, 9, 14–17], individuals with documented SARS-CoV-2 infection recorded in the national COVID-19 registry within 300 days of dengue infection or enrollment as a population-based control were excluded.

### Inclusion and Exclusion Criteria (Acute)

Singaporean citizens/permanent residents age ≥18 years with complete demographic data and those who did not die before enrollment were enrolled for comparison of excess health care utilization from T_0_ to T_0_ + 30 between dengue patients and population-based controls. A flowchart of the cohort construction is provided below in Figure 1.

### Inclusion and Exclusion Criteria (Postacute)

**Figure 2.**
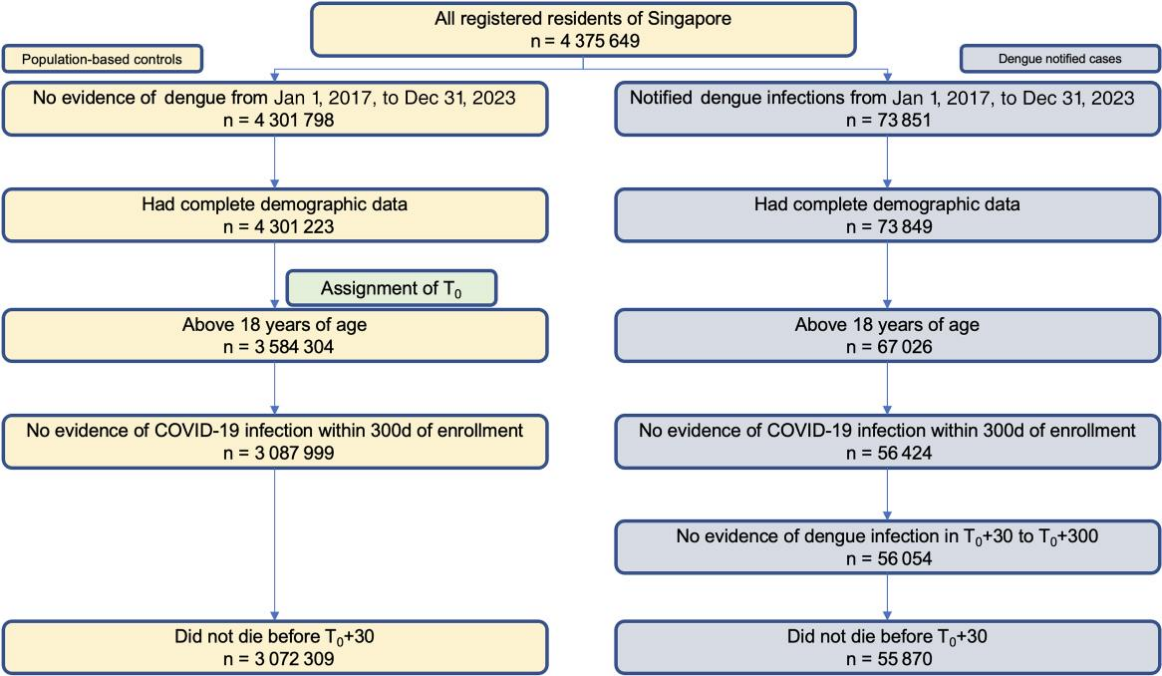
Cohort construction flowchart for comparisons of excess health care utilization in T_0_ + 31 to T_0_ + 300 between dengue patients and population-based controls. Abbreviation: COVID-19, coronavirus disease 2019.

For post-acute comparisons, the primary change was that dengue-infected individuals should have no evidence of reinfection in the postacute period and those who did not die within the first 30 days after the date of enrollment. Only individuals with 1 dengue infection were included due to the small percentage of individuals with multiple notified dengue infections (>3%). Therefore, to provide clean estimates of the excess health care utilization following 1 dengue infection vs population-based controls, individuals with reinfections were excluded. For both acute and postacute comparisons, only Singapore citizens/permanent residents were included as administrative claims databases used health care bills for this group, as they were enrolled in the compulsory government administered medical savings scheme (see “Outcomes”). A flowchart of the cohort construction for postacute comparisons is provided below in Figure 2.

### Outcomes

The outcomes of interest were total excess acute and postacute hospital utilization in the 30 days following T_0_ and 31 to 300 days following T_0_, respectively. We broke down total hospital utilization as (1) all-cause inpatient hospital utilization—length of stay in inpatient settings or the number of unique hospital inpatient admissions, (2) all-cause intensive care unit utilization—length of stay in the intensive care unit (ICU) or the number of unique ICU admissions, (3) all-cause emergency department utilization—the total number of unique ED visits. We also examined (4) total costs incurred in inpatient settings and (5) excess mortality in the acute/postacute periods. ED, hospital, and ICU utilization were assessed using the national health care claims database.

Health care utilization was assessed using the national health care claims database. In the study setting, enrollment in the national government-administered medical savings scheme (Medisave) is compulsory; Medisave can be billed for inpatient care and outpatient treatment at both public/private health care providers [18]. This enabled comprehensive capture of health care utilization across all care settings (inpatient admissions, ICU admissions, ED visits). Costs incurred are defined as unsubdisized charges. At public health care providers, costs are computed as a sum of out-of-pocket costs incurred by the patient, costs claimed through Medisave, and the government subsidies provided for treatment and care. Costs at private health care providers comprised out-of-pocket costs incurred by the patient and costs claimed through Medisave.

### Covariates

We accounted for differences in baseline characteristics by incorporating the following covariates: demographics (age, sex, ethnicity), number of COVID-19 vaccination doses (0/1, 2, 3+), comorbidity burden (Charlson Comorbidity Index [CCI]), socioeconomic status (SES), ED attendance in the past 5 years, and inpatient admissions in the past 5 years. SES was classified by housing type, a key marker of SES in Singapore [19].

### Statistical Analysis

Baseline sociodemographic characteristics of the test-positives and respective population-based controls, along with standardized mean differences (SMDs) between groups, were computed for each period in which different variants circulated. We implemented 2-part models to ascertain health care utilization and health care costs for dengue patients vs population-based controls. In each 2-part model, we accounted for imbalances in sociodemographic/comorbidity status in the exposed and unexposed groups by propensity score weighting. To do so, logistic regressions were trained, taking the outcome variable as dengue exposure and explanatory variables as COVID-19 vaccination status, age, ethnicity, sex, socioeconomic status, previous hospital utilization, and comorbidity status. We then used estimated propensity scores to obtain overlap weights, which were defined as equal to the propensity score and 1 – propensity score.

For the 2-part model, the first part of the model takes a binomial link function to estimate the probability of any emergency department visit, inpatient hospitalization, ICU visit, or hospitalization cost incurred in the follow-up period (T_0_ to T_0_ + 30, or T_0_ + 31 to T_0_ + 300). This enabled us to estimate excess risks in terms of odds ratios (ORs) and to quantify the increase/decrease in acute/postacute risk of hospital utilization/incurring of hospital costs in dengue patients vs population-based controls. In the second part of the model, we used generalized linear models with the Gamma link function to estimate the (1) excess length of stay or number of unique visits in dengue patients among individuals with any inpatient admissions, (2) excess length of stay or number of unique visits in dengue patients among individuals with any ICU admissions, (3) number of unique ED visits in dengue patients among individuals with any ED admissions, (4) excess costs in dengue patients vs population-based controls, conditioned on individuals who incurred any health care costs.

For (1)–(3), we expressed the excess utilization of unique visits/length of stay in dengue patients as a rate ratio, which we defined as the ratio of estimated rates of unique visits/length of stay in dengue patients vs population-based controls who had any ED/inpatient/ICU utilization. We also expressed excess utilization of unique visits/length of stay in dengue patients in terms of excess burdens, which we defined as the estimated difference in utilization between dengue patients vs population-based controls among those with any ED/inpatient/ICU utilization. For (4), we similarly defined excess hospital costs in dengue patients as a rate ratio, which we defined as the estimated ratio between the amount of hospital costs incurred in dengue patients vs controls among those who incurred any hospital costs. We also expressed excess health care costs (US$1 = SG$1.34) in dengue patients in terms of excess burdens, which we defined as the estimated difference in health care costs in dengue patients vs controls who incurred any health care costs. The observed follow-up time was included as offsets in both components of the 2-part model.

To examine effect modification, we conducted analyses in subgroups by age (18–65, 65+), comorbidities (CCI = 0, 1+), number of previous hospitalizations (0, 1+), sex (male or female), and time period (pre-2020, post-2020).

In the sensitivity analysis, we explored the use of inverse probability weighting, stabilized inverse probability weighting, and a doubly robust approach where the same explanatory variables used to estimate propensity scores were additionally adjusted for in the outcome regressions to prevent model misspecification in the propensity score models and outcome models. These were done to ascertain the influence of weights on our estimates of excess health care utilization in both the acute and postacute phases. We additionally stress-tested our analytical framework using negative-outcome controls. Negative controls can be utilized to detect suspected/unsuspected sources of spurious bias and may lessen concerns about unmeasured confounding and other latent biases in outcome ascertainment [20]. For example, if there was a difference in the risk of health care utilization in the comparator groups, the ascertainment bias can extend to that of negative-outcome controls; successful testing of the outcome control therefore alleviates concerns about biases in the analytical framework and underlying data. We took asthma as the negative-outcome control and reran our entire analytical framework on examining whether dengue exposure leads to increased risk of new-incident asthma over the follow-up period. The only difference was that competing-risk regressions were utilized instead of 2-part models. There should be no causal relation between the exposure (dengue infection) and the risk of the negative-outcome control (asthma).

### Ethics Statement

This study was done as part of national public health research under the Infectious Diseases Act, Singapore; separate ethics review by an institutional review board was not required.

## RESULTS

### Study Population

In total, 3 072 971 population-based controls and 56 417 dengue cases were enrolled for acute phase analysis when inclusion and exclusion criteria were met (Figure 1), and a corresponding 3 072 309 population-based controls and 55 870 dengue cases were enrolled for postacute phase analysis (Figure 2). Baseline sociodemographic and clinical characteristics of infected individuals and population-based controls after propensity score weighting are presented in Table 1. After weighting, differences in demographic characteristics, socioeconomic status, and comorbidity between the 2 groups were small, with all SMDs >0.01 (Table 1). Among dengue cases, the majority (≥68.96%) had mild disease and did not have dengue-linked hospitalizations, which were defined as hospitalizations within 30 days of being notified of a dengue diagnosis.

**Table 1.** Summary Statistics for Dengue Cases and Population-Based Controls for Acute and Postacute Comparisons of Excess Health Care Utilization After Overlap Weights Were Employed

	Acute			Postacute		
	Population-Based Controls (Weighted) (n = 2 221 231), No. (%)	Dengue Cases (Weighted) (n = 50 185), No. (%)	SMD (Weighted)	Population-Based Controls (Weighted) (n = 2 221 231), No. (%)	Dengue Cases (Weighted) (n = 50 185), No. (%)	SMD (Weighted)
Age, mean (SD)	48.66 (18.22)	48.66 (17.85)	>0.01	48.54 (18.19)	48.54 (17.78)	>0.01
CCI, mean (SD)	0.35 (1.15)	0.35 (1.12)	>0.01	0.34 (1.12)	0.34 (1.09)	>0.01
No. of previous hospitalizations, mean (SD)	1.37 (4.33)	1.37 (2.44)	>0.01	1.35 (4.27)	1.35 (2.4)	>0.01
Male	29 446 (53.43)	29 446 (53.43)	>0.01	29 137 (53.37)	29 137 (53.37)	>0.01
Ethnicity						
Chinese	43 694 (79.28)	43 694 (79.28)	>0.01	43 297 (79.31)	43 297 (79.31)	>0.01
Indian	4572 (8.29)	4572 (8.29)	>0.01	4515 (8.27)	4515 (8.27)	>0.01
Malay	5205 (9.44)	5205 (9.44)	>0.01	5147 (9.43)	5147 (9.43)	>0.01
Others	1642 (2.98)	1642 (2.98)	>0.01	1634 (2.99)	1634 (2.99)	>0.01
Housing type						
1–2 rooms	2140 (3.88)	2140 (3.88)	>0.01	2093 (3.83)	2093 (3.83)	>0.01
3 rooms	7106 (12.89)	7106 (12.89)	>0.01	7004 (12.83)	7004 (12.83)	>0.01
4 rooms	14 023 (25.44)	14 023 (25.44)	>0.01	13 891 (25.44)	13 891 (25.44)	>0.01
5 rooms/exec	21 405 (38.84)	21 405 (38.84)	>0.01	21 257 (38.94)	21 257 (38.94)	>0.01
Others	370 (0.67)	370 (0.67)	>0.01	360 (0.66)	360 (0.66)	>0.01
Private	10 071 (18.27)	10 071 (18.27)	>0.01	9988 (18.3)	9988 (18.3)	>0.01
COVID-19 vaccination status at T_0_						
Boosted	15 081 (27.36)	15 081 (27.36)	>0.01	14 902 (27.3)	14 902 (27.3)	>0.01
Fully vaccinated	1800 (3.27)	1800 (3.27)	>0.01	1751 (3.21)	1751 (3.21)	>0.01
Partially vaccinated	38 232 (69.37)	38 232 (69.37)	>0.01	37 941 (69.5)	37 941 (69.5)	>0.01

SMD <0.1 indicates good balance between exposure groups.

Abbreviations: CCI, Charlson Comorbidity Index; COVID-19, coronavirus disease 2019; SMD, standardized mean difference.

### Increased Risk of Acute and Postacute Health Care Utilization in Dengue Patients

There was an increased risk of emergency department visits, inpatient visits, and incurring any hospitalization bill in the 0–30-day (acute) and 31–300-day (postacute) periods following dengue infection vs population-based controls (Table 2). In the acute period, there were (1) a 17.589 times greater risk of incurring any hospital bills, (2) a 38.989 times higher risk of emergency department admission, (3) a 17.599 times greater risk of inpatient admission, and (4) a 9.59 times greater risk of ICU admission for dengue patients vs population-based controls. In the postacute period, there was a ∼20% greater risk of incurring any hospital bill, utilizing the emergency department, or having any inpatient/ICU admission in dengue patients vs population-based controls.

**Table 2.** Risk and Rates of Health Care Utilization and Costs Among Dengue Patients vs Population-Based Controls in the Acute and Postacute Periods

		Acute			
Comparison	Outcome	Odds Ratio (95% CI)^a^	Rate Ratio (95% CI)^b^	Excess Burden per Person (95% CI)^c^	Dengue Patients With Counts >0
Dengue vs population-based controls	Total bill^d^	17.587 (16.635–18.608)*	10.324 (8.216–13.884)*	1206.57 (1171.34–1241.80)*	17 714
Dengue vs population-based controls	ED visits^e^	38.989 (36.309–41.93)*	25.571 (19.31–37.838)*	0.442 (0.436–0.448)*	20 501
Dengue vs population-based controls	Inpatient visits^f^	17.599 (16.645–18.621)*	11.938 (10.308–14.179)*	0.315 (0.31–0.319)*	17 699
Dengue vs population-based controls	Inpatient LoS^f^	17.599 (16.645–18.621)*	15.852 (11.868–23.861)*	1.433 (1.4–1.466)*	17 699
Dengue vs population-based controls	ICU visits^g^	9.59 (6.454–14.942)*	17.906 (−7.909 to 4.197)	0.008 (0.006–0.01)*	238
Dengue vs population-based controls	ICU LOS^g^	9.59 (6.454–14.942)*	18.218 (−8.323 to 4.347)	0.025 (0.021–0.03)*	238

		Postacute			
Comparison	Outcome	Odds Ratio (95% CI)	Rate Ratio (95% CI)^b^	Excess Burden per 270 Person-Days (95% CI)^c^	Dengue Patients With Counts >0
Dengue vs population-based controls	Total bill^d^	1.269 (1.227–1.312)*	1.244 (1.2–1.29)*	75.50 (63.29–87.70)*	9100
Dengue vs population-based controls	ED visits^e^	1.257 (1.209–1.307)*	1.285 (1.235–1.338)*	0.012 (0.01–0.014)*	6316
Dengue vs population-based controls	Inpatient visits^f^	1.269 (1.227–1.312)*	1.157 (1.123–1.192)*	0.01 (0.008–0.012)*	9084
Dengue vs population-based controls	Inpatient LoS^f^	1.269 (1.227–1.312)*	1.339 (1.291–1.39)*	0.08 (0.07–0.089)*	9084
Dengue vs population-based controls	ICU visits^g^	1.253 (1.036–1.518)*	1.31 (1.052–1.666)*	0 (0–0.001)*	247
Dengue vs population-based controls	ICU LOS^g^	1.253 (1.036–1.518)*	1.235 (0.989–1.567)	0.001 (0–0.002)	247

Abbreviations: ED, emergency department; ICU, intensive care unit; LOS, length of stay.

*P = .05.

^a^Odds ratio estimated from the first part of the 2-part model. An odds ratio >1 represents increased risk of any ED visits/inpatient admissions/ICU admissions or incurring any hospital costs.

^b^Excess proportion of (1) emergency department visits among dengue patients vs comparator control group who had any ED visits, (2) unique inpatient episodes and inpatient length of stay among dengue patients vs comparator control group who had any inpatient admissions, (3) ICU inpatient episodes and ICU length of stay among dengue patients vs comparator control group who had any ICU admissions, (4) costs among dengue patients vs comparator control group who had any hospital costs incurred. Any value >1 represents higher utilization levels among dengue patients vs comparator group. (1 – rate ratio)*100 is the percent increase in utilization in the test-positive group.

^c^Excess burden of (1) emergency department visits among dengue patients vs comparator control group who had any ED visits, (2) unique inpatient episodes and inpatient length of stay among dengue patients vs comparator control group who had any inpatient admissions, (3) ICU inpatient episodes and ICU length of stay among dengue patients vs comparator control group who had any ICU admissions, (4) costs among dengue patients vs comparator control group who had any hospital costs incurred. Any value >0 represents higher utilization levels among dengue patients vs comparator group.

^d^Risks of incurring hospital costs and excess rates/burdens of costs among dengue patients vs comparator group who had any hospital costs incurred.

^e^Excess risk/rate/burden of emergency department visits in dengue patients vs comparator control group.

^f^Excess risk/rate/burden of unique inpatient episodes and inpatient length of stay among test-positives vs population-based controls who had any inpatient admissions.

^g^Excess number of ICU inpatient episodes and ICU length of stay among test-positives vs population-based controls who had any inpatient admissions.

### Increased Risk of Excess Health Care Utilization in the Acute Period of Dengue Infection

Among individuals who had any ED visits, there was a 25.571-fold (rate ratio [RR], 25.571; 95% CI, 19.31–37.838) (Table 2) higher rate of unique emergency department visits in dengue patients vs population-based controls. Similarly, among patients who had any inpatient admissions, dengue patients were associated with higher rates of unique inpatient visits and longer lengths of stay vs population-based controls. In particular, dengue patients had a 15.852-fold (RR, 15.852; 95% CI, 11.868–23.861) (Table 2) greater length of stay vs population-based controls.

Overall, risk of incurring any hospital bills was also increased in dengue patients vs population-based controls (OR, 17.587; 95% CI, 16.635–18.608). Among those who incurred any hospital bills, bill sizes were on average 10.324 times (RR, 10.324; 95% CI, 8.216–13.884) higher in dengue patients vs population-based controls. This translated to a US$1206.57 (95% CI, US$1171.34–US$1241.80) increase in average bill sizes for dengue patients vs population-based controls among those who incurred any hospital bills. In total, excess total bill sizes over the study period incurred by dengue patients in the acute phase were around US$21 363 084.

### Increased Risk of Excess Health Care Utilization in the Postacute Period of Dengue Infection

We found that dengue patients were associated with higher postacute excess health care utilization vs population-based controls, but these estimates were lower compared with excess utilization in the acute period (Table 2). Specifically, in the postacute period spanning 31 to 300 days after enrollment, individuals with dengue infection had 1.285-fold (RR, 1.285; 95% CI, 1.235–1.338) (Table 2) higher risk of unique ED visits vs population-based controls among those who had any ED visits. Among those who had inpatient admissions, we found elevated utilization in dengue patients, with 1.157 times higher unique inpatient visits (RR, 1.157; 95% CI, 1.123–1.192) and 1.339 times higher inpatient length of stay (RR, 1.339; 95% CI, 1.291–1.39) in the postacute period vs population-based controls. There was also an estimated US$75.50 (95% CI, US$63.29–US$87.70) increase in average bill sizes in dengue patients vs population-based controls among those who incurred any hospital bills. In total, excess total bill sizes over the study period incurred by dengue patients in the postacute phase were around US$687 032.

### Subgroup Analysis

We repeated our analysis in subgroups of patients based on number of past hospitalizations (0, 1+), period of enrollment (pre-COVID: 2017–2019; post-COVID: 2020–2023 eras), comorbidity status (CCI = 0, 1+), and age group (18–65, 65+). Across all subgroups, we found elevated risks of unique ED visits, inpatient admission, ICU admissions, and incurring any hospital bill in dengue patients vs population-based controls. Elevated risks persisted across both the acute and postacute phases of infection, with higher risks of health care utilization in the acute phase vs postacute phase. Across all subgroups, in dengue patients, rates and excess burdens of unique ED visits were higher among all individuals who had any ED visits vs the comparator group of population-based controls (Supplementary Table 1).

Similarly, rates and excess burdens of inpatient admissions and inpatient length of stay were higher among individuals who had any inpatient admissions vs population-based controls. In both the acute and postacute periods, the excess burden of health care costs, unique ED/inpatient visits, and inpatient/ICU length of stay was higher in dengue patients who were age ≥65 years, had a CCI score ≥1, or had ≥1 past hospitalization among individuals who utilized these health care resources (Supplementary Data).

### Sensitivity Analyses

We explored alternative weighting schemes for our study (inverse probability weights/stabilized inverse probability weights). These weighting schemes resulted in extreme weights and standardized mean differences that indicated that balance in covariates was not achieved between exposure groups. These skewed estimates of excess health care utilization, and we therefore relied on overlapping weighting to present results on excess health care utilization. There was no increased risk of asthma (negative-outcome control) post–dengue infection—this alleviated concerns that our study design and analytical plan may have possibly generated spurious estimates of health care (Supplementary Tables 2–6, Supplementary Figure 1).

## DISCUSSION

Our study found increased risk of health care utilization in dengue patients vs population-based controls both in the acute period of initial infection (0–30 days postinfection) and in the postacute period (31–300 days following dengue infection) in a population-based multi-ethnic adult cohort, where the majority of dengue patients did not require hospitalization. Excess health care utilization and health care costs were higher in dengue patients who were age ≥65 years and patients who had prior comorbidities.

Increased health care utilization in the postacute phase following dengue infection is likely attributable to nonresolving symptom persistence and chronic sequelae experienced after dengue in a subset of survivors; this has been documented in small prospective cohorts and large retrospective population-based cohort studies [2–7]. Chronic sequelae following not just COVID-19 but also hospitalizations for other infective syndromes, such as influenza and sepsis, have been described in the literature as being attributable to the physiological stress of acute hospitalization [21, 22]. Notably, despite the vast majority of our cohort being comprised of dengue-infected individuals with mild infection not requiring initial hospitalization, increased health care utilization was still observed in the postacute phase, with a modest associated excess cost to the health system of ∼US$75.498 over the postacute period of observation in dengue survivors vs population-based controls. Given rising numbers of dengue infections driven by various factors, including climate change [23], even a small increase in costs attributable to chronic sequelae of dengue in a fraction of survivors may potentially translate into a disproportionate burden on health care systems in under-resourced tropical LMICs. Yet, our estimates suggest that the burdens incurred on the health system were far greater in the acute phase as compared with the postacute phase. This is in contrast to a prior Mexican study that utilized prior estimates of symptom persistence post-dengue to evaluate the impact on the economic burden attributable to dengue, where chronic symptom persistence following dengue infection was estimated to lead to a 43% increase in disability-adjusted life-years attributable to dengue over prior estimates [24]. A subgroup analysis also indicated that older adults had far greater increased health care utilization following dengue; this is consistent with elevated risk of poorer outcomes in elderly dengue survivors and those with increased comorbidity burdens compared with their younger counterparts [25]. As changing epidemiological trends result in a shift of the disease burden of dengue toward older age groups, surveillance for nonresolving symptoms and chronic sequelae should be prioritized in populations at risk of poorer dengue outcomes.

Our study has several strengths. We used large, nationally representative electronic health databases, combined with individually resolved surveillance data, to estimate the postacute excess risks and rates of hospital utilization for dengue patients across a long study period. We examined for each outcome, not only the risk but also excess burdens due to dengue, which enabled quantification of risk on the absolute scale. We examined the use of alternative weighting schemes and were able to replicate the increased health care utilization using these alternative methods. Our analysis using negative-outcome controls reduced the possibility that our analytical framework produced spurious estimates of excess health care utilization. The estimates provided by this study could be used to further ascertain the cost-effectiveness of dengue prevention programs in the study setting.

This study has several limitations: (1) Despite widespread availability of rapid-test kits across health care settings and a comprehensive case and virus surveillance system in the study setting, the unexposed groups may have been contaminated by undiagnosed or asymptomatic dengue infections [26]; while we used comprehensive national notification databases to reduce the likelihood of misclassification, misclassification would result in more conservative estimates of excess health care utilization in the dengue group. (2) While our study comprised a nationally representative multi-ethnic Asian population, it was predominantly of Chinese ethnicity, which may limit the generalizability of the study findings to other dengue-endemic regions with more ethnically diverse populations. (3) Only adults age ≥18 years were included, restricting generalizability to pediatric populations. (4) Health care claims data might under-report estimates of milder sequelae not affecting reimbursement. Health care claims data also do not comprise data from private health care; our estimates of health care utilization, though based on a comprehensive health care claims database with national-level coverage, may still be an underestimate of the morbidity attributable to dengue infection. (5) The study was conducted in a high-income nation with a relatively high health care standard. This may have influenced care for dengue patients and biased estimates of acute and postacute health care utilization following dengue infection conservatively. (6) As administrative claims databases do not have information on the clinical severity of dengue cases, subgroup analyses by dengue severity could not be conducted. Future work should seek to estimate excess health care utilizations following dengue infection by clinical severity. (7) While our analyses balanced a substantial number of sociodemographic and socioeconomic covariates between comparator groups, future work that includes a test-negative control group may help improve estimation of excess health care utilization by better accounting for health care–seeking behavior.

## CONCLUSION

There is increased excess risk and rates of health care utilization in the 300 days post–dengue infection when compared with contemporary population-based controls.

## Supplementary Material

ofaf401_Supplementary_Data

## Supplementary Data

Supplementary materials are available at *Open Forum Infectious Diseases* online. Consisting of data provided by the authors to benefit the reader, the posted materials are not copyedited and are the sole responsibility of the authors, so questions or comments should be addressed to the corresponding author.
