# Supplementary material for: Acute and Postacute Health Care Utilization and Costs After Dengue Infection: A Population-Based Cohort Study
Source: Open Forum Infect Dis. 2025 Jul 3;12(7):ofaf401. doi: 10.1093/ofid/ofaf401 (PMC12287693; doi:10.1093/ofid/ofaf401)
Supplement: ofaf401_Supplementary_Data [file ofaf401_supplementary_data.docx]

| Dengue vs population-based controls | | | | | | | |
| --- | --- | --- | --- | --- | --- | --- | --- |
|  |  | Acute | | | Post-Acute | | |
| Strata | Outcome | Odds Ratio (95% CI)^&^ | Rate Ratio (95% CI) ^&&^ | Excess Burden Per Person (95% CI) ^&&&^ | Odds Ratio (95% CI) ^&^ | Rate Ratio (95% CI) ^&&^ | Excess Burden Per Person (95% CI) ^&&&^ |
| 0 hospitalisations | Total bill^#^ | 25.883 (22.424, 30.066)* | 14.641 (9.316, 34.153)* | 466.217 (445.635, 486.798)* | 1.3 (1.215, 1.39)* | 1.326 (1.236, 1.426)* | 125.654 (95.144, 156.165)* |
| 0 hospitalisations | ED visits^##^ | 67.977 (58.574, 79.458)* | 56.026 (-814.069, 27.081) | 0.414 (0.406, 0.422)* | 1.193 (1.108, 1.285)* | 1.258 (1.17, 1.356)* | 0.018 (0.013, 0.024)* |
| 0 hospitalisations | Inpatient visits^###^ | 25.938 (22.468, 30.133)* | 21.36 (13.368, 53.109)* | 0.165 (0.16, 0.17)* | 1.298 (1.213, 1.389)* | 1.273 (1.194, 1.36)* | 0.023 (0.017, 0.03)* |
| 0 hospitalisations | Inpatient LoS^###^ | 25.938 (22.468, 30.133)* | 30.726 (15.692, 731.973)* | 0.526 (0.509, 0.543)* | 1.298 (1.213, 1.389)* | 1.073 (0.998, 1.154) | 0.016 (0, 0.032) |
| 0 hospitalisations | ICU visits^####^ | 12.718 (4.287, 60.834)* | 12.824 (-3.741, 2.354) | 0.001 (0.001, 0.002)* | 1.318 (0.83, 2.112) | 1.57 (0.98, 3.117) | 0.001 (0, 0.002) |
| 0 hospitalisations | ICU LOS^####^ | 12.718 (4.287, 60.834)* | 9.843 (-3.642, 2.077) | 0.004 (0.002, 0.005)* | 1.318 (0.83, 2.112) | 0.979 (0.568, 1.604) | 0 (-0.003, 0.002) |
| 1+ hospitalisations | Total bill^#^ | 18.462 (17.36, 19.651)* | 9.759 (7.62, 13.566)* | 1809.607 (1748.004, 1871.21)* | 1.139 (1.095, 1.184)* | 1.262 (1.211, 1.315)* | 454.114 (375.905, 532.324)* |
| 1+ hospitalisations | ED visits^##^ | 30.159 (27.837, 32.733)* | 19.181 (14.512, 28.279)* | 0.466 (0.458, 0.475)* | 1.169 (1.116, 1.224)* | 1.331 (1.27, 1.396)* | 0.074 (0.063, 0.086)* |
| 1+ hospitalisations | Inpatient visits^###^ | 18.463 (17.36, 19.653)* | 10.864 (9.401, 12.864)* | 0.437 (0.43, 0.445)* | 1.14 (1.096, 1.185)* | 1.179 (1.142, 1.218)* | 0.063 (0.051, 0.075)* |
| 1+ hospitalisations | Inpatient LoS^###^ | 18.463 (17.36, 19.653)* | 14.629 (10.768, 22.805)* | 2.172 (2.113, 2.231)* | 1.14 (1.096, 1.185)* | 1.415 (1.355, 1.48)* | 0.571 (0.505, 0.638)* |
| 1+ hospitalisations | ICU visits^####^ | 9.449 (6.188, 15.234)* | 18.963 (-6.708, 3.927) | 0.014 (0.011, 0.016)* | 1.261 (1.023, 1.558)* | 1.293 (1, 1.719)* | 0.002 (0, 0.004)* |
| 1+ hospitalisations | ICU LOS^####^ | 9.449 (6.188, 15.234)* | 19.639 (-7.07, 4.109) | 0.043 (0.034, 0.052)* | 1.261 (1.023, 1.558)* | 1.357 (1.049, 1.817)* | 0.01 (0.002, 0.018)* |
| After 2020 | Total bill^#^ | 15.822 (14.829, 16.899)* | 9.915 (7.747, 13.762)* | 1219.025 (1178.413, 1259.636)* | 1.316 (1.266, 1.368)* | 1.368 (1.314, 1.425)* | 429.074 (376.653, 481.494)* |
| After 2020 | ED visits^##^ | 35.278 (32.457, 38.423)* | 24.679 (17.835, 40.048)* | 0.409 (0.402, 0.416)* | 1.301 (1.243, 1.362)* | 1.374 (1.311, 1.441)* | 0.058 (0.05, 0.066)* |
| After 2020 | Inpatient visits^###^ | 15.832 (14.838, 16.911)* | 10.814 (9.193, 13.128)* | 0.292 (0.287, 0.298)* | 1.315 (1.265, 1.367)* | 1.201 (1.161, 1.243)* | 0.049 (0.04, 0.059)* |
| After 2020 | Inpatient LoS^###^ | 15.832 (14.838, 16.911)* | 14.465 (10.667, 22.459)* | 1.405 (1.367, 1.443)* | 1.315 (1.265, 1.367)* | 1.463 (1.403, 1.528)* | 0.417 (0.374, 0.46)* |
| After 2020 | ICU visits^####^ | 9.438 (6.06, 15.604)* | 19.643 (-6.817, 4.022) | 0.01 (0.008, 0.012)* | 1.357 (1.091, 1.692)* | 1.451 (1.142, 1.91)* | 0.002 (0.001, 0.004)* |
| After 2020 | ICU LOS^####^ | 9.438 (6.06, 15.604)* | 19.304 (-7.129, 4.099) | 0.029 (0.023, 0.036)* | 1.357 (1.091, 1.692)* | 1.535 (1.188, 2.08)* | 0.009 (0.004, 0.015)* |
| Before 2020 | Total bill^#^ | 22.432 (20.208, 24.972)* | 10.085 (7.979, 13.698)* | 1160.594 (1123.859, 1197.33)* | 9.101 (4.227, 23.832)* | 13.074 (-4.902, 2.796) | -27.594 (-85.571, 30.383) |
| Before 2020 | ED visits^##^ | 45.883 (40.392, 52.37)* | 22.302 (15.729, 38.309)* | 0.53 (0.519, 0.541)* | 1.157 (1.083, 1.236)* | 0.972 (0.917, 1.031) | -0.018 (-0.031, -0.006)* |
| Before 2020 | Inpatient visits^###^ | 22.459 (20.231, 25.004)* | 12.097 (9.668, 16.153)* | 0.37 (0.361, 0.379)* | 1.155 (1.071, 1.245)* | 0.905 (0.843, 0.971)* | -0.028 (-0.042, -0.014)* |
| Before 2020 | Inpatient LoS^###^ | 22.459 (20.231, 25.004)* | 14.238 (10.462, 22.275)* | 1.479 (1.436, 1.521)* | 1.158 (1.084, 1.238)* | 0.892 (0.841, 0.946)* | -0.088 (-0.137, -0.039)* |
| Before 2020 | ICU visits^####^ | 9.101 (4.227, 23.832)* | 9.248 (-6.27, 2.65) | 0.003 (0.002, 0.004)* | 1.158 (1.084, 1.238)* | 0.896 (0.842, 0.952)* | 0 (-0.001, 0.002) |
| Before 2020 | ICU LOS^####^ | 22.432 (20.208, 24.972)* | 10.085 (7.979, 13.698)* | 1160.594 (1123.859, 1197.33)* | 1.086 (0.735, 1.609) | 1.11 (0.75, 1.688) | 0 (-0.004, 0.004) |
| CCMI=0 | Total bill^#^ | 21.91 (20.421, 23.54)* | 13.245 (9.675, 20.984)* | 984.281 (953.193, 1015.369)* | 1.233 (1.184, 1.283)* | 1.171 (1.123, 1.223)* | 124.769 (91.441, 158.098)* |
| CCMI=0 | ED visits^##^ | 55.295 (50.383, 60.849)* | 37.926 (24.979, 78.736)* | 0.429 (0.423, 0.436)* | 1.207 (1.15, 1.268)* | 1.219 (1.163, 1.279)* | 0.022 (0.017, 0.027)* |
| CCMI=0 | Inpatient visits^###^ | 21.953 (20.459, 23.588)* | 15.417 (12.547, 19.99)* | 0.285 (0.281, 0.29)* | 1.232 (1.183, 1.283)* | 1.095 (1.055, 1.135)* | 0.016 (0.009, 0.022)* |
| CCMI=0 | Inpatient LoS^###^ | 21.953 (20.459, 23.588)* | 26.471 (15.152, 104.607)* | 1.148 (1.114, 1.182)* | 1.232 (1.183, 1.283)* | 1.182 (1.126, 1.242)* | 0.08 (0.057, 0.103)* |
| CCMI=0 | ICU visits^####^ | 12.347 (6.86, 24.98)* | 19.004 (-5.288, 3.395) | 0.004 (0.003, 0.005)* | 1.285 (0.973, 1.703) | 1.273 (0.907, 1.872) | 0.001 (0, 0.002) |
| CCMI=0 | ICU LOS^####^ | 12.347 (6.86, 24.98)* | 16.155 (-5.894, 3.404) | 0.011 (0.008, 0.013)* | 1.285 (0.973, 1.703) | 1.609 (1.128, 2.576)* | 0.005 (0.001, 0.008)* |
| CCMI=1+ | Total bill^#^ | 12.008 (10.927, 13.218)* | 7.347 (5.497, 11.064)* | 2457.513 (2318.107, 2596.92)* | 1.358 (1.272, 1.45)* | 1.382 (1.302, 1.469)* | 1300.074 (1072.709, 1527.44)* |
| CCMI=1+ | ED visits^##^ | 18.27 (16.281, 20.566)* | 11.162 (8.325, 16.929)* | 0.516 (0.498, 0.535)* | 1.391 (1.296, 1.493)* | 1.441 (1.35, 1.542)* | 0.205 (0.171, 0.24)* |
| CCMI=1+ | Inpatient visits^###^ | 11.965 (10.887, 13.172)* | 7.088 (5.969, 8.719)* | 0.477 (0.461, 0.493)* | 1.36 (1.274, 1.452)* | 1.248 (1.192, 1.308)* | 0.161 (0.128, 0.194)* |
| CCMI=1+ | Inpatient LoS^###^ | 11.965 (10.887, 13.172)* | 9.214 (7.132, 13.009)* | 3.025 (2.912, 3.139)* | 1.36 (1.274, 1.452)* | 1.508 (1.426, 1.598)* | 1.633 (1.427, 1.839)* |
| CCMI=1+ | ICU visits^####^ | 8.142 (4.787, 15.063)* | 18.292 (-4.822, 3.155) | 0.03 (0.022, 0.038)* | 1.304 (1.002, 1.703)* | 1.445 (1.087, 2.026)* | 0.007 (0.002, 0.013)* |
| CCMI=1+ | ICU LOS^####^ | 8.142 (4.787, 15.063)* | 20.536 (-5.1, 3.406) | 0.107 (0.079, 0.135)* | 1.304 (1.002, 1.703)* | 1.117 (0.831, 1.513) | 0.008 (-0.014, 0.03) |
| Age 18–65 | Total bill^#^ | 20.382 (18.972, 21.928)* | 10.75 (8.05, 16.17)* | 926.037 (892.048, 960.026)* | 1.221 (1.172, 1.273)* | 1.121 (1.073, 1.171)* | 96.093 (59.318, 132.867)* |
| Age 18–65 | ED visits^##^ | 47.617 (43.524, 52.222)* | 32.067 (22.079, 58.556)* | 0.425 (0.418, 0.431)* | 1.208 (1.15, 1.268)* | 1.227 (1.166, 1.293)* | 0.027 (0.02, 0.034)* |
| Age 18–65 | Inpatient visits^###^ | 20.398 (18.986, 21.946)* | 13.936 (11.441, 17.82)* | 0.272 (0.267, 0.277)* | 1.221 (1.172, 1.273)* | 1.091 (1.051, 1.133)* | 0.016 (0.009, 0.022)* |
| Age 18–65 | Inpatient LoS^###^ | 20.398 (18.986, 21.946)* | 18.212 (12.277, 35.252)* | 1.003 (0.974, 1.032)* | 1.221 (1.172, 1.273)* | 1.178 (1.121, 1.238)* | 0.085 (0.059, 0.11)* |
| Age 18–65 | ICU visits^####^ | 9.836 (5.658, 18.852)* | 15.805 (-5.115, 3.102) | 0.004 (0.003, 0.006)* | 1.221 (0.927, 1.612) | 1.289 (0.967, 1.782) | 0.001 (0, 0.002) |
| Age 18–65 | ICU LOS^####^ | 9.836 (5.658, 18.852)* | 14.755 (-6.091, 3.333) | 0.013 (0.01, 0.016)* | 1.221 (0.927, 1.612) | 1.104 (0.788, 1.566) | 0.001 (-0.003, 0.005) |
| Age 65+ | Total bill^#^ | 15.494 (14.155, 16.989)* | 8.318 (5.969, 13.703)* | 2334.708 (2200.838, 2468.578)* | 1.461 (1.375, 1.552)* | 1.533 (1.449, 1.626)* | 1287.579 (1130.272, 1444.886)* |
| Age 65+ | ED visits^##^ | 25.987 (23.105, 29.334)* | 15.461 (10.924, 26.432)* | 0.517 (0.502, 0.533)* | 1.469 (1.371, 1.575)* | 1.431 (1.344, 1.527)* | 0.136 (0.113, 0.159)* |
| Age 65+ | Inpatient visits^###^ | 15.489 (14.149, 16.985)* | 8.714 (7.261, 10.89)* | 0.49 (0.476, 0.503)* | 1.46 (1.374, 1.552)* | 1.271 (1.213, 1.332)* | 0.141 (0.114, 0.168)* |
| Age 65+ | Inpatient LoS^###^ | 15.489 (14.149, 16.985)* | 12.808 (9.015, 22.103)* | 3.215 (3.096, 3.333)* | 1.46 (1.374, 1.552)* | 1.564 (1.479, 1.657)* | 1.348 (1.195, 1.501)* |
| Age 65+ | ICU visits^####^ | 9.569 (5.533, 18.18)* | 19.242 (-4.995, 3.286) | 0.023 (0.017, 0.03)* | 1.418 (1.084, 1.864)* | 1.512 (1.139, 2.123)* | 0.006 (0.002, 0.01)* |
| Age 65+ | ICU LOS^####^ | 9.569 (5.533, 18.18)* | 14.085 (-5.457, 3.071) | 0.076 (0.055, 0.097)* | 1.418 (1.084, 1.864)* | 1.587 (1.156, 2.363)* | 0.025 (0.009, 0.042)* |

SI Table 1: Risk and rates of healthcare utilisation and costs among dengue patients versus population-based controls or COVID-19 patients in the acute and post-acute period across subgroups. Numbers in parentheses refer to 95% confidence intervals. Overlap weights were employed in balancing exposure groups.

^&^Odds ratio estimated from the first part of the two part model. An odds ratio > 1 represents increased risk of any ED visits/inpatient admissions/ICU admissions or incurring of any hospital costs.

^#^Risks of incurring hospital costs, and excess rates/burdens of costs among dengue patients versus comparator group whom had any hospital costs incurred

^##^Excess risk/rate/burden of emergency department visits in dengue patients versus comparator control group

^###^Excess risk/rate/burden of unique inpatient episodes and inpatient length of stay among test-positives versus test-negatives whom had any inpatient admissions

^####^Excess number of ICU inpatient episodes and ICU length of stay among test-positives versus test-negatives whom had any inpatient admissions

^&&^Excess proportion of (**1**) emergency department visits among dengue patients versus comparator control group whom had any ED visits (**2**) unique inpatient episodes and inpatient length of stay among dengue patients versus comparator control group whom had any inpatient admissions (**3**) ICU inpatient episodes and ICU length of stay among dengue patients versus comparator control group whom had any ICU admissions (**4**) costs of among dengue patients versus comparator control group whom had any hospital costs incurred. Any value >1 represents higher utilisation levels among dengue patients versus comparator group

^&&&^Excess burden of (**1**) emergency department visits among dengue patients versus comparator control group whom had any ED visits (**2**) unique inpatient episodes and inpatient length of stay among dengue patients versus comparator control group whom had any inpatient admissions (**3**) ICU inpatient episodes and ICU length of stay among dengue patients versus comparator control group whom had any ICU admissions (**4**) costs of among dengue patients versus comparator control group whom had any hospital costs incurred. Any value >0 represents higher utilisation levels among dengue patients versus comparator group

(1- Rate Ratio)*100 is the percentage increase in utilisation in the test-positive group.

Statistical significance at *0.05 , **0.01,***0.001 level.

| Subgroup | HR | 95% CI, LB | 95% CI, UB | EB | 95% CI, LB | 95% CI, UB | Controls | Controls with outcome (%) | Dengue | Dengue with outcome (%) | p-value |
| --- | --- | --- | --- | --- | --- | --- | --- | --- | --- | --- | --- |
| All | 0.930 | 0.346 | 2.497 | 0.000 | 0.000 | 0.000 | 3092055.000 | 287 (0.009) | 49755.000 | 4 (0.008) | 0.885 |
| Chinese | 1.106 | 0.352 | 3.472 | 0.000 | 0.000 | 0.000 | 2301803.000 | 162 (0.007) | 39649.000 | 3 (0.008) | 0.863 |
| Non Chinese | 0.543 | 0.076 | 3.883 | 0.000 | -0.001 | 0.001 | 792165.000 | 140 (0.018) | 10106.000 | 1 (0.01) | 0.543 |
| Age 18-40 | - | - | - | 0.000 | -0.001 | 0.000 | 1098705.000 | 68 (0.006) | 17873.000 | 0 (0) | - |
| Age 41-60 | 0.665 | 0.093 | 4.783 | 0.000 | -0.001 | 0.000 | 1090904.000 | 97 (0.009) | 18147.000 | 1 (0.006) | 0.685 |
| Age 61+ | 1.246 | 0.397 | 3.913 | 0.000 | -0.001 | 0.001 | 904110.000 | 156 (0.017) | 13735.000 | 3 (0.022) | 0.706 |
| CCMI 0 | 0.707 | 0.098 | 5.077 | 0.000 | 0.000 | 0.000 | 2691212.000 | 103 (0.004) | 42610.000 | 1 (0.002) | 0.730 |
| CCMI 1+ | 1.010 | 0.323 | 3.163 | 0.000 | -0.002 | 0.002 | 400201.000 | 183 (0.046) | 7145.000 | 3 (0.042) | 0.986 |
| 1-3 room flats | - | - | - | 0.000 | -0.001 | 0.000 | 609723.000 | 91 (0.015) | 8076.000 | 0 (0) | - |
| 4-5 room flats | 1.183 | 0.440 | 3.181 | 0.000 | -0.001 | 0.001 | 2163893.000 | 236 (0.011) | 31575.000 | 4 (0.013) | 0.739 |
| Private property | - | - | - | 0.000 | -0.001 | 0.000 | 228995.000 | 10 (0.004) | 9787.000 | 0 (0) | - |
| Female | - | - | - | 0.000 | -0.001 | 0.000 | 1600994.000 | 178 (0.011) | 23171.000 | 0 (0) | - |
| Male | 2.031 | 0.749 | 5.511 | 0.000 | 0.000 | 0.001 | 1490527.000 | 120 (0.008) | 26584.000 | 4 (0.015) | 0.164 |
| Hospitalized | 1.103 | 0.353 | 3.445 | 0.000 | 0.000 | 0.000 | 3027886.000 | 282 (0.009) | 34314.000 | 3 (0.009) | 0.865 |
| Not hospitalized | 0.640 | 0.090 | 4.563 | 0.000 | -0.001 | 0.001 | 3252859.000 | 309 (0.009) | 15441.000 | 1 (0.006) | 0.656 |
| 2017–2019 | 0.590 | 0.147 | 2.373 | 0.000 | -0.001 | 0.000 | 2933417.000 | 306 (0.01) | 35131.000 | 2 (0.006) | 0.457 |
| 2020–2023 | 1.702 | 0.423 | 6.841 | 0.000 | -0.001 | 0.001 | 3589868.000 | 298 (0.008) | 14624.000 | 2 (0.014) | 0.454 |

SI Table 2: Hazards ratios (HR) and excess burdens (EB) per 1000 for the negative-outcome control (asthma) in the post-acute followup period of 30 to 300 days for dengue-infected individuals versus controls with accompanying 95% confidence intervals (CI), in the main cohort and all subgroups.

HR>1 denotes higher risk of pre-specified sequelae and EB>0 denotes higher incidence of pre-specified sequelae in dengue infected individuals versus controls.

HRs are estimated using competing risk regression taking death as the competing risk with overlap weights applied and EBs were computed by taking the differences in weighted incidences between comparator groups. *Denotes statistical significance at 95% level. **Denotes p values for HRs less than 0.0017 (i.e the Bonferonni-corrected cut-off)˙.

- not estimable due to small number of outcomes in either comparator group

| Variable | Population-based controls (N = 3072971) | Dengue infected (N = 56417) | SMD | Population-based controls (weighted) (N = 47) | Dengue infected (Weighted) (N = 48192) | SMD (weighted) |
| --- | --- | --- | --- | --- | --- | --- |
| Chinese | 2291788 (74.58%) | 44789 (79.39%) | 0.11 | 2871501 (78.33%) | 2297596 (74.72%) | 0.09 |
| Indian | 285824 (9.30%) | 4666 (8.27%) | 0.04 | 290558 (7.93%) | 285363 (9.28%) | 0.05 |
| Malay | 374497 (12.19%) | 5290 (9.38%) | 0.09 | 381368 (10.4%) | 369718 (12.02%) | 0.05 |
| Others | 120862 (3.93%) | 1672 (2.96%) | 0.05 | 122535 (3.34%) | 122238 (3.98%) | 0.03 |
| Male | 1479576 (48.15%) | 30203 (53.54%) | 0.11 | 2046238 (55.82%) | 1501036 (48.82%) | 0.14 |
| 1-2 Room | 143286 (4.66%) | 2179 (3.86%) | 0.04 | 146455 (3.99%) | 142349 (4.63%) | 0.03 |
| 3-Room | 458369 (14.92%) | 7230 (12.82%) | 0.06 | 465636 (12.7%) | 454715 (14.79%) | 0.06 |
| 4-Room | 968743 (31.52%) | 14246 (25.25%) | 0.14 | 1517546 (41.4%) | 964870 (31.38%) | 0.22 |
| 5-Room/Exec | 1202163 (39.12%) | 21815 (38.67%) | 0.01 | 1224890 (33.41%) | 1213429 (39.46%) | 0.12 |
| Others | 77176 (2.51%) | 372 (0.66%) | 0.15 | 77550 (2.12%) | 68167 (2.22%) | 0.01 |
| Private | 223234 (7.26%) | 10575 (18.74%) | 0.35 | 233886 (6.38%) | 231384 (7.52%) | 0.03 |
| COVID-19 Boosted | 827723 (26.94%) | 15425 (27.34%) | 0.01 | 844841 (23.05%) | 858494 (27.92%) | 0.11 |
| COVID-19 Fully vaccinated | 106205 (3.46%) | 1841 (3.26%) | 0.01 | 642543 (17.53%) | 108277 (3.52%) | 0.78 |
| COVID-19 Unvaccinated/partially vaccinated | 2139043 (69.61%) | 39151 (69.40%) | 0 | 2178578 (59.43%) | 2108144 (68.56%) | 0.2 |
| Age | 49.07 (18.01) | 48.66 (17.86) | 0.02 | 49.79 (16.74) | 49.02 (17.64) | 0.04 |
| CCMI | 0.28 (0.94) | 0.36 (1.13) | 0.07 | 1.26 (2.53) | 0.31 (0.98) | 0.92 |
| Previous hospitalisations | 0.83 (1.91) | 1.39 (2.54) | 0.25 | 33.45 (78.81) | 1.12 (1.8) | 14.4 |

SI Table 3: Summary statistics for dengue cases and population-based controls for acute comparisons of excess healthcare utilisation after inverse-probability weights were employed. SMDs refer to standardized mean differences, with SMD<0.1 indicating good balance between exposure groups.

| Variable | Population-based controls (N = 3072971) | Dengue infected (N = 56417) | SMD | Population-based controls (weighted) (N = 47) | Dengue infected (Weighted) (N = 48192) | SMD (weighted) |
| --- | --- | --- | --- | --- | --- | --- |
| Chinese | 2291788 (74.58%) | 44789 (79.39%) | 0.11 | 2819733 (78.33%) | 41421 (74.72%) | 0.09 |
| Indian | 285824 (9.30%) | 4666 (8.27%) | 0.04 | 285319 (7.93%) | 5145 (9.28%) | 0.05 |
| Malay | 374497 (12.19%) | 5290 (9.38%) | 0.09 | 374493 (10.4%) | 6665 (12.02%) | 0.05 |
| Others | 120862 (3.93%) | 1672 (2.96%) | 0.05 | 120326 (3.34%) | 2204 (3.98%) | 0.03 |
| Male | 1479576 (48.15%) | 30203 (53.54%) | 0.11 | 2009348 (55.82%) | 27061 (48.82%) | 0.14 |
| 1-2 Room | 143286 (4.66%) | 2179 (3.86%) | 0.04 | 143815 (3.99%) | 2566 (4.63%) | 0.03 |
| 3-Room | 458369 (14.92%) | 7230 (12.82%) | 0.06 | 457242 (12.7%) | 8198 (14.79%) | 0.06 |
| 4-Room | 968743 (31.52%) | 14246 (25.25%) | 0.14 | 1490187 (41.4%) | 17395 (31.38%) | 0.22 |
| 5-Room/Exec | 1202163 (39.12%) | 21815 (38.67%) | 0.01 | 1202807 (33.41%) | 21876 (39.46%) | 0.12 |
| Others | 77176 (2.51%) | 372 (0.66%) | 0.15 | 76152 (2.12%) | 1229 (2.22%) | 0.01 |
| Private | 223234 (7.26%) | 10575 (18.74%) | 0.35 | 229669 (6.38%) | 4171 (7.52%) | 0.03 |
| COVID-19 Boosted | 827723 (26.94%) | 15425 (27.34%) | 0.01 | 829610 (23.05%) | 15477 (27.92%) | 0.11 |
| COVID-19 Fully vaccinated | 106205 (3.46%) | 1841 (3.26%) | 0.01 | 630960 (17.53%) | 1952 (3.52%) | 0.78 |
| COVID-19 Unvaccinated/partially vaccinated | 2139043 (69.61%) | 39151 (69.40%) | 0 | 2139302 (59.43%) | 38006 (68.56%) | 0.2 |
| Age | 49.07 (18.01) | 48.66 (17.86) | 0.02 | 49.79 (16.74) | 49.02 (17.64) | 0.04 |
| CCMI | 0.28 (0.94) | 0.36 (1.13) | 0.07 | 1.26 (2.53) | 0.31 (0.98) | 0.92 |
| Previous hospitalisations | 0.83 (1.91) | 1.39 (2.54) | 0.25 | 33.45 (78.81) | 1.12 (1.8) | 14.4 |

SI Table 4: Summary statistics for dengue cases and population-based controls for acute comparisons of excess healthcare utilisation after stabilised inverse-probability weights were employed. SMDs refer to standardized mean differences, with SMD<0.1 indicating good balance between exposure groups.

| Variable | Population-based controls (N = 3072971) | Dengue infected (N = 56417) | SMD | Population-based controls (weighted) (N = 47) | Dengue infected (Weighted) (N = 48192) | SMD (weighted) |
| --- | --- | --- | --- | --- | --- | --- |
| Chinese | 2291289 (74.58%) | 44371 (79.42%) | 0.12 | 2783723 (77.81%) | 2297720 (74.69%) | 0.07 |
| Indian | 285775 (9.30%) | 4607 (8.25%) | 0.04 | 290444 (8.12%) | 285982 (9.3%) | 0.04 |
| Malay | 374398 (12.19%) | 5229 (9.36%) | 0.09 | 381045 (10.65%) | 369966 (12.03%) | 0.04 |
| Others | 120847 (3.93%) | 1663 (2.98%) | 0.05 | 122511 (3.42%) | 122554 (3.98%) | 0.03 |
| Male | 1479246 (48.15%) | 29877 (53.48%) | 0.11 | 1958562 (54.74%) | 1501076 (48.8%) | 0.12 |
| 1-2 Room | 143214 (4.66%) | 2130 (3.81%) | 0.04 | 146245 (4.09%) | 142593 (4.64%) | 0.03 |
| 3-Room | 458210 (14.91%) | 7124 (12.75%) | 0.06 | 465368 (13.01%) | 454817 (14.78%) | 0.05 |
| 4-Room | 968560 (31.53%) | 14109 (25.25%) | 0.14 | 1430401 (39.98%) | 965357 (31.38%) | 0.19 |
| 5-Room/Exec | 1202020 (39.12%) | 21661 (38.77%) | 0.01 | 1224487 (34.23%) | 1213569 (39.45%) | 0.11 |
| Others | 77158 (2.51%) | 362 (0.65%) | 0.15 | 77522 (2.17%) | 68772 (2.24%) | 0.01 |
| Private | 223147 (7.26%) | 10484 (18.76%) | 0.35 | 233701 (6.53%) | 231115 (7.51%) | 0.03 |
| COVID-19 Boosted | 827322 (26.93%) | 15236 (27.27%) | 0.01 | 844079 (23.59%) | 858166 (27.9%) | 0.1 |
| COVID-19 Fully vaccinated | 106074 (3.45%) | 1789 (3.20%) | 0.01 | 555538 (15.53%) | 108485 (3.53%) | 0.67 |
| COVID-19 Unvaccinated/partially vaccinated | 2138913 (69.62%) | 38845 (69.53%) | 0 | 2178106 (60.88%) | 2109571 (68.58%) | 0.17 |
| Age | 49.06 (18.01) | 48.54 (17.79) | 0.03 | 49.68 (16.92) | 49.01 (17.6) | 0.04 |
| CCMI | 0.28 (0.94) | 0.34 (1.10) | 0.06 | 1.12 (2.39) | 0.31 (0.98) | 0.8 |
| Previous hospitalisations | 0.83 (1.90) | 1.37 (2.49) | 0.24 | 28.83 (73.89) | 1.11 (1.79) | 12.5 |

SI Table 5: Summary statistics for dengue cases and population-based controls for post-acute comparisons of excess healthcare utilisation after inverse-probability weights were employed. SMDs refer to standardized mean differences, with SMD<0.1 indicating good balance between exposure groups.

| Variable | Population-based controls (N = 3072971) | Dengue infected (N = 56417) | SMD | Population-based controls (weighted) (N = 47) | Dengue infected (Weighted) (N = 48192) | SMD (weighted) |
| --- | --- | --- | --- | --- | --- | --- |
| Chinese | 2291289 (74.58%) | 44371 (79.42%) | 0.12 | 2734005 (77.81%) | 41038 (74.69%) | 0.07 |
| Indian | 285775 (9.30%) | 4607 (8.25%) | 0.04 | 285257 (8.12%) | 5108 (9.3%) | 0.04 |
| Malay | 374398 (12.19%) | 5229 (9.36%) | 0.09 | 374239 (10.65%) | 6608 (12.03%) | 0.04 |
| Others | 120847 (3.93%) | 1663 (2.98%) | 0.05 | 120323 (3.42%) | 2189 (3.98%) | 0.03 |
| Male | 1479246 (48.15%) | 29877 (53.48%) | 0.11 | 1923582 (54.74%) | 26810 (48.8%) | 0.12 |
| 1-2 Room | 143214 (4.66%) | 2130 (3.81%) | 0.04 | 143633 (4.09%) | 2547 (4.64%) | 0.03 |
| 3-Room | 458210 (14.91%) | 7124 (12.75%) | 0.06 | 457056 (13.01%) | 8123 (14.78%) | 0.05 |
| 4-Room | 968560 (31.53%) | 14109 (25.25%) | 0.14 | 1404854 (39.98%) | 17242 (31.38%) | 0.19 |
| 5-Room/Exec | 1202020 (39.12%) | 21661 (38.77%) | 0.01 | 1202618 (34.23%) | 21675 (39.45%) | 0.11 |
| Others | 77158 (2.51%) | 362 (0.65%) | 0.15 | 76137 (2.17%) | 1228 (2.24%) | 0.01 |
| Private | 223147 (7.26%) | 10484 (18.76%) | 0.35 | 229527 (6.53%) | 4128 (7.51%) | 0.03 |
| COVID-19 Boosted | 827322 (26.93%) | 15236 (27.27%) | 0.01 | 829003 (23.59%) | 15327 (27.9%) | 0.1 |
| COVID-19 Fully vaccinated | 106074 (3.45%) | 1789 (3.20%) | 0.01 | 545616 (15.53%) | 1938 (3.53%) | 0.67 |
| COVID-19 Unvaccinated/partially vaccinated | 2138913 (69.62%) | 38845 (69.53%) | 0 | 2139205 (60.88%) | 37677 (68.58%) | 0.17 |
| Age | 49.06 (18.01) | 48.54 (17.79) | 0.03 | 49.68 (16.92) | 49.01 (17.6) | 0.04 |
| CCMI | 0.28 (0.94) | 0.34 (1.10) | 0.06 | 1.12 (2.39) | 0.31 (0.98) | 0.8 |
| Previous hospitalisations | 0.83 (1.90) | 1.37 (2.49) | 0.24 | 28.83 (73.89) | 1.11 (1.79) | 12.5 |

SI Table 6: Summary statistics for dengue cases and population-based controls for post-acute comparisons of excess healthcare utilisation after stabilised inverse-probability weights were employed. SMDs refer to standardized mean differences, with SMD<0.1 indicating good balance between exposure groups.

SI Figure 1: Distribution of weights for acute/post-acute comparisons between population-based controls and dengue infected groups, using overlap weights (OW), inverse probability weights (IPW) and stabilised inverse probability weights (SIPW).
